# Microwave-Assisted Improved Synthesis of Oxazolidin-2-ones, Oxazolidine-2-thiones and Thiazolidine-2-thione Chiral Auxiliaries

**DOI:** 10.3390/molecules16108803

**Published:** 2011-10-20

**Authors:** Rosmarbel Morales-Nava, Mario Fernández-Zertuche, Mario Ordóñez

**Affiliations:** Centro de Investigaciones Químicas, Universidad Autónoma del Estado de Morelos, Ave. Universidad 1001, Col. Chamilpa, Cuernavaca, Morelos, 62209, Mexico; Email: rmn@uaem.mx (R.M.N.)

**Keywords:** microwave, oxazolidin-2-ones, oxazolidine-2-thiones, thiazolidine-2-thiones

## Abstract

A microwave assisted method for the synthesis of some typical 4-substituted oxazolidinone chiral auxiliaries used in asymmetric synthesis is reported in this work. Under these conditions, treatment of (*S*)-phenylalaninol, (*S*)-phenylglycinol, (*S*)-valinol and (1*S*, 2*R*)-norephedrine with ethyl carbonate or carbon disulfide under the appropriate and specific microwave reaction conditions, led to an efficient synthesis of some oxazolidin-2-ones, oxazolidine-2-thiones and thiazolidine-2-thiones. The methodology reported in this paper provides these chiral auxiliaries with improved yields and a remarkable reduction on the reaction times, particularly in the case of thiazolidine-2-thiones, as compared with the conventional methods. All the auxiliaries prepared here show spectroscopic data in full agreement with those previously reported in the literature.

## 1. Introduction

Chiral auxiliaries such as the classical Evans oxazolidin-2-ones [1,2,3] have been widely used in the synthesis of natural products and pharmacologically active compounds [4]. During the last decade, however, the corresponding sulfur analogues, the oxazolidine-2-thiones and thiazolidine-2-thiones [5,6] have also become popular chiral auxiliaries in asymmetric synthesis since they can be equally or even more effective as chiral inductors and the fact that after the chiral transformation has been achieved, their removal is much easier to accomplish than that of the original oxazolidin-2-one systems. The preparation of the 4-substituted oxazolidin-2-one chiral auxiliaries is usually carried out in a two-step sequence of reactions; reduction of an amino acid [7,8,9,10] to the corresponding amino alcohol, followed by cyclization of the amino alcohol to the oxazolidin-2-one system by treatment with ethyl carbonate [11,12], and phosgene or phosgene derivatives for long reaction times [13,14]. Oxazolidine-2-thiones and thazolidine-2-thiones can also be obtained from the same amino alcohols precursors by condensation with thiophosgene [15] or carbon disulfide [16,17]. In the latter method with carbon disulfide, oxazolidine-2-thiones require mild conditions (CS_2_ and a limited reaction time); but thiazolidine-2-thiones require more drastic conditions such as excess CS_2_, basic media and heating over extended periods of time, from 16 to 24 hours.

The use of microwave methodologies in organic synthesis has attracted considerable attention in recent years [18]. We wish to report here a microwave-assisted method for the synthesis of oxazolidin-2-ones, oxazolidine-2-thiones and thiazolidine-2-thiones from amino alcohols which results in shortened reaction times and improved yields of these chiral auxiliaries.

## 2. Results and Discussion

**Scheme 1:**
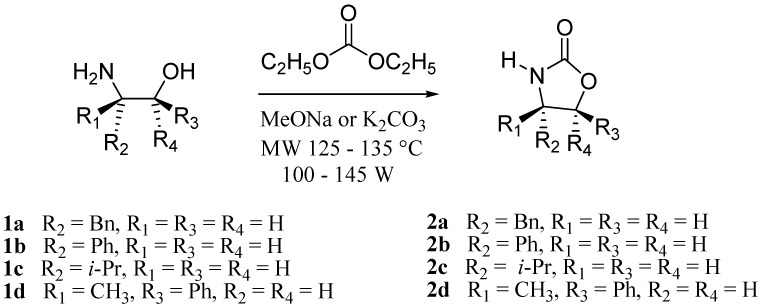
Synthesis of oxazolidin-2-ones.

In our method, the 4-substituted oxazolidin-2-ones **2a-d** were readily available from the corresponding amino alcohols **1a-d** as shown in Scheme 1. Formation of **2a-d** was achieved under microwave irradiation (125–135 °C) of **1a-d** in the presence of diethyl carbonate and sodium methoxide or K_2_CO_3_ [11,12].

Analysis of the data in Table 1 shows that in all cases, the yields obtained under microwave irradiation conditions are better than the yields reported in the literature for the conventional methods. Moreover, the reaction times required to achieve full conversion to the corresponding oxazolidin-2-ones are dramatically diminished in comparison to the reaction times required with the conventional methods. At this point, it was of prime importance to verify their optical rotation and calculate the enantiomeric or diastereomeric excess. This was accomplished by comparing the specific rotations of oxazolidin-2-ones **2a-d** with the specific rotations reported in the literature [11,16].

**Table 1:** Reaction conditions in the synthesis of oxazolidin-2-ones.

Compound	% Yield MW	% Yield Conventional Method	MW Reaction Time (minutes)	Conventional Reaction Time (minutes)	Reaction Conditions MW	% e.e. MW
2a *	96	82–85	20	480	135 °C, 100 W	99
2b **	98	80–85	20	60	125 °C, 125 W	96
2c *	94	84–86	20	480	135 °C, 125 W	95
2d *	87	68–74	15	90	135 °C, 145 W	96 ^a^

* Reaction carried out with sodium methoxide; ** Reaction carried out with potassium carbonate; ^a^ Related to the diastereomeric excess.

**Scheme 2:**
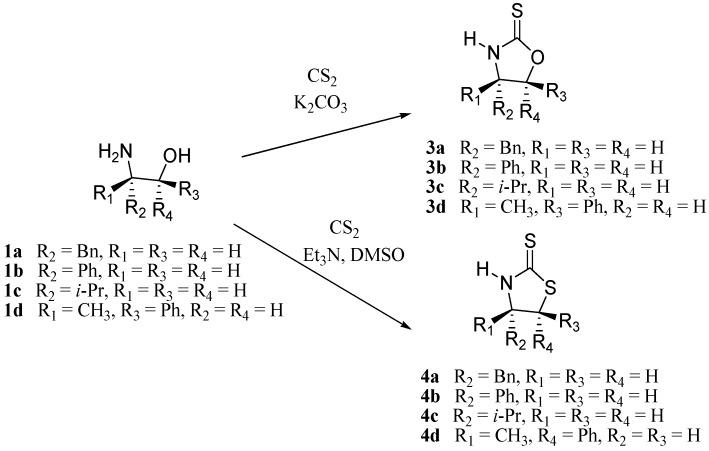
Synthesis of oxazolidine-2-thiones and thiazolidine-2-thiones.

After establishing the optimal conditions for the synthesis of the oxazolidin-2-one systems, we turned our attention to the corresponding sulfur analogs **3a-d** and **4a-d**. We explored several reaction conditions by varying solvents, equivalents of carbon disulfide and temperature ranges. For example, when the reactions are carried out in toluene, dichloromethane, hexane or THF the yields are very low and the reaction times to achieve full conversion to the products were higher than those reported in the literature. Temperature was a key issue, and a temperature range between 120 °C–150 °C is required for the preparation of oxazolidin-2-ones, temperatures below 55 °C for oxazolidine-2-thiones and temperatures around 100 °C for thiazolidine-2-thiones. Regarding the equivalents of carbon disulfide, initially we used 5.0 equivalents since this is the amount of equivalents reported in the conventional methods, but in our method we found that 3.0 equivalents yielded the best results. Thus, microwave irradiation of amino alcohols **1a-d** with carbon disulfide in a basic medium can lead to the formation of either the oxazolidine-2-thiones **3a-d** or the thermodynamically favored thiazolidine-2-thiones **4a-d** regardless of the amount of carbon disulfide used (Scheme 2). This initial behavior under microwave conditions shows some analogy to the work of Wu [16] on the conventional synthesis of these chiral auxiliaries.

After exploring several reaction conditions such as different solvents, equivalents of carbon disulfide and temperatures, we found a set of reaction conditions to carry out the selective formation of either **3a-d** or **4a-d** with reduced reaction times, particularly in the case of the thiazolidine-2-thiones **4a-d** [17]. Specifically, microwave irradiation (50 W) of **1a-d** in the presence of carbon disulfide and potassium carbonate at 50 °C lead to the exclusive formation of the oxazolidine-2-thiones **3a-d** in a very pure form and in nearly quantitative yields (Table 2), in these cases, 0 minutes means that the reactions essentially occur instantaneously. Although the % yields and reaction times achieved for **3a** and **3b** in this method do not offer any major advantages over the conventional method, it is noteworthy that **3c** is obtained in a better yield than the conventional method [17]. On the other hand, microwave irradiation of **1a-d** with carbon disulfide and in the presence of DMSO as a solvent, led now to the formation of the thiazolidine-2-thione chiral auxiliaries **4a-d**.

**Table 2:** Reaction conditions used in the synthesis of chiral oxazolidine-2-thiones and thiazolidine-2-thiones.

Compound	% Yield MW	% Yield Conventional Methods	MW Reaction Time (minutes)	Conventional Reaction Time (minutes)	Reaction Conditions MW	% e.e.
**3a**	99	99	0	0	K_2_CO_3_, CS_2_, 50 °C, 50 W	98
**3b**	99	99	0	0	K_2_CO_3_, CS_2_, 50 °C, 50 W	97
**3c**	89	62–64	10	20	K_2_CO_3_, CS_2_, 50 °C, 50 W	90
**3d**	82	70–72	10	60	K_2_CO_3_, CS_2_, 50 °C, 50 W	98^a^
**4a**	92	80–82	60	960	CS_2_, DMSO, 100 °C, 40 W	98
**4b**	89	77–78	90	960	CS_2_, DMSO, 100 °C, 40 W	99
**4c**	90	80–84	110	960	CS_2_, DMSO, 110 °C, 100 W	94
**4d**	65	55–60	120	960	CS_2_, DMSO, 110 °C, 100 W	93 ^a^

^a^ Related to the diastereomeric excess.

As it can be seen in Table 2, formation of the chiral auxiliaries **4a-d** under these conditions show a remarkable reduction in the reaction times required to achieve full conversion of **1a-d** to these systems as compared to the conventional method [11]. Compounds **3a-d** and **4a-d** were characterized by ^1^H- and ^13^C-NMR spectroscopy as well as optical rotation; their recorded data are in full agreement with previously reported values. The results on Table 2 also show that the rate of the cyclization reaction is dependent on the nature of R_3_ and R_4_. When R_3_ = R_4_ = H, (**4c**) the reaction proceeds smoothly and affords yields around 90%. However, when R_3_ = Ph, (**4d**) the cyclization still occurs, but in a lower yield [11].

**Scheme 3:**
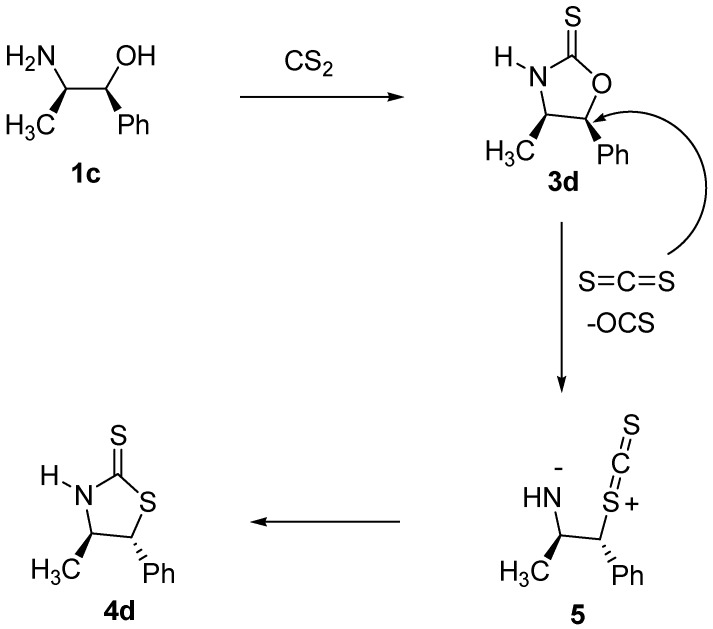
Mechanism for the formation of thiazolidine-2-thiones.

On the other hand, since formation of **4d** proceeds with inversion of configuration at C-5, the reaction mechanism involved in the formations of the thiazolidine-2-thiones deserves some comment. In carrying out the synthesis of **3d**, we observed that when the reaction temperature exceeded 50 °C (70 to 80 °C) the formation of **4d** began and TLC showed the gradual disappearance of **3d**. We propose a mechanism (Scheme 3) in which **3d** may be an intermediate in the formation of **4d**. Nucleophilic attack by carbon disulfide on **3d** to afford **5** would explain the observed inversion of configuration at C-5 in analogy to the mechanism proposed by Le Corre and co-workers in 1995 [17]. From **5**, ring closure leads to the formation of **4d**. The fact that inversion of configuration at C-5 occurs on **3d** during this process, was confirmed by analysis of the coupling constants of the hydrogens attached to C-4 and C-5 on **3d** and **4d**. In compound **3d** these hydrogens show a coupling constant of 8.4 Hz, which is consistent with a *cis* arrangement. On the other hand, in compound **4d** these hydrogens show a coupling constant of 8.8 Hz, consistent with a *trans* arrangement. These changes on the coupling constants are similar to closely related compounds reported in the literature, as well as other spectroscopic data [19,20,21,22,23,24].

## 3. Experimental

### 3.1. General

The microwave experiments were carried out in a CEM Focused Microwave Discover reactor. The reactions were carried out under the OpenVessel and BenchMate modalities using the glassware and magnetic stirrers supplied by the equipment manufacturer. The reaction conditions were determined using in most cases the same reagents and ratios as the ones reported in the conventional methods with appropriate adjustments in the amount of equivalents used. The reactions were made with quantities of 0.5 g to 1.0 g of amino alcohols. The power and temperature ranges used, were derived after several trials monitoring the % conversion of starting materials to products. All solvents were used after distillation at normal pressure. ^1^H-NMR spectra were recorded at 400 and 200 MHz with CDCl_3_ as solvent and tetramethylsilane as internal standard. ^13^C-NMR spectra were recorded at 100 and 50 MHz with CDCl_3_ as solvent using a Varian Inova 400 or a Mercury 200 instrument. Optical rotations were measured on a Perkin-Elmer 241 polarimeter at room temperature.

### 3.2. Synthesis of Oxazolidin-2-ones **2a-d**

The equivalents of amino alcohols **1a-d**, base and ethyl carbonate were almost the same as the ones reported in the literature [1,2,3]. The reagents were placed in a 10 mL vessel in the BenchMate mode. The products were purified by column chromatography with a hexane-ethyl acetate solvent system.

*(S)-4-Benzyl-1,3-oxazolidin-2-one *(**2a**). The aminoalcohol **1a** (1.00 g, 6.61 mmol, 1.0 eq.), diethyl carbonate (1.17 g, 1.2 mL, 9.92 mmol, 1.5 eq.) and sodium methoxide (0.017 g, 0.33 mmol, 0.05 eq.) were placed in a 10 mL vessel in the microwave system and reacted under the BenchMate modality during 20 minutes at 135 °C and 100 W of power. When the reaction was completed, the resulting mixture was partitioned with a mixture of CH_2_Cl_2_ (15 mL) and H_2_O (20 mL). The organic layer was separated and the aqueous layer was extracted with CH_2_Cl_2_ (2 × 15 mL). The organic extracts were dried over Na_2_SO_4_ and the solvent evaporated to furnish a residue which was purified through a silica gel chromatographic column eluting with hexane-ethyl acetate (3:2) to give 1.12 g (96% yield) of **2a** as a slightly yellow solid. The spectroscopic data were compared with those reported in the literature [10,11]: m. p. = 84.5–86.5 °C (Lit. = 87–88 °C), [α]_D_ = −62.5 c = 1 in CHCl_3_ ([α]_D(Lit.)_ = −63 c = 1 in CHCl_3_), e.e. = 99%. ^1^H-NMR (400 MHz, CDCl_3_): 7.36–7.17 (m, 5H), 5.69 (br, s, 1H), 4.45 (t, 1H, *J* = 8.4 Hz), 4.15 (t, 1H, *J* = 8.4 Hz), 4.09 (m, 1H), 2.88 (d, 2H, *J *= 7.2 Hz). ^13^C-NMR (100 MHz, CDCl_3_): 159.3, 127.4, 69.8, 54.0, 41.7 Anal. Calcd. for C_10_H_11_NO_2_: C, 67.77; H, 6.20; N, 7.90. Found: C, 67.50; H, 6.13; N, 7.71.

*(S)-4-Phenyl-1,3-oxazolidin-2-one* (**2b**). The aminoalcohol **1b** (1.00 g, 7.29 mmol, 1.0 eq.), diethyl carbonate (1.80 g, 1.8 mL, 15.31 mmol, 2.1 eq.) and potassium carbonate (0.15 g, 1.09 mmol, 0.15 eq.) of were placed in a 10 mL vessel in the microwave system and reacted under the BenchMate modality for 20 minutes at 125 °C and 125 W of power. When the reaction was completed, the resulting mixture was dissolved in CH_2_Cl_2_ (30 mL) and the insoluble K_2_SO_3_ were filtered off with suction and brine (15 mL) was added to the organic phase. The organic layer was separated and the aqueous layer was extracted with CH_2_Cl_2_ (2 × 15 mL). The organic extracts were dried over Na_2_SO_4_ and the solvent evaporated to furnish a residue which was purified through a silica gel chromatographic column eluting with hexane-ethyl acetate (3:2) to give 1.16 g (98% yield) of **2b** as a white solid. The spectroscopic data were compared with those reported in the literature [12]: m. p. = 131–133 °C (Lit. = 129–132 °C), [α]_D_ = + 46.1 c=2 in CHCl_3_ ([α]_D(Lit.)_ = +48 c=2 in CHCl_3_), e.e. = 96%. ^1^H-NMR (200 MHz, CDCl_3_): 7.33–7.23 (m, 5H), 6.44 (br, s, 1H), 4.88 (t, 1H, *J* = 7.7 Hz), 4.64 (t, 1H, *J* = 8.6 Hz), 4.08 (td, 1H, *J *= 8.2, 1.2 Hz. ^13^C-NMR (50 MHz, CDCl_3_): 160.4, 139.6, 129.2, 128.7, 126.0, 72.6, 56.5 Anal. Calcd. for C_9_H_9_NO_2_: C, 66.27; H, 5.50; N, 8.58. Found: C, 66.15; H, 5.80; N, 8.64.

*(S)-4-Isopropyl-1,3-oxazolidin-2-one* (**2c**). The aminoalcohol **1c** (1.00 g, 9.69 mmol, 1.0 eq.), diethyl carbonate (1.71 g, 1.7 mL, 14.54 mmol, 1.5 eq.)and sodium methoxide (0.026 g, 0.48 mmol, 0.05 eq.) were placed in a 10 mL vessel in the microwave system and reacted under the BenchMate modality for 20 minutes at 135 °C and 125 W of power. When the reaction was completed, the resulting mixture was partitioned with a mixture of CH_2_Cl_2_ (10 mL) and H_2_O (20 mL). The organic layer was separated and the aqueous layer was extracted with CH_2_Cl_2_ (2 × 10 mL). The organic extracts were dried over Na_2_SO_4_ and the solvent evaporated to furnish a residue which was purified through a silica gel chromatographic column eluting with hexane-ethyl acetate (3:2) to give 1.17 g (94% yield) of **2c** as a slightly yellow solid. The spectroscopic data were compared with those reported in the literature [13]: m. p. = 71.5–73 °C (Lit. = 70–73 °C), [α]_D_ = −18 c = 6 in EtOH ([α]_D(Lit.)_ = −17.0 c = 6 in EtOH), e.e. = 95%. ^1^H-NMR (400 MHz, CDCl_3_): 7.18 (br, s, 1H), 4.44 (dd, 1H, *J* = 8.8, 8.8 Hz), 4.10 (dd, 1H, *J* = 8.8, 6.8 Hz), 3.62 (dddd, 1H, *J* = 8.8, 6.8, 2.4, 0.8 Hz), 1.72 (dh, 1H, *J* = 6.8, 6.8 Hz), 0.96 (d, 3H, *J *= 6.8 Hz), 0.90 (d, 3H, *J *= 6.8 Hz). ^13^C-NMR (100 MHz, CDCl_3_): 160.0, 68.6, 58.6, 32.8, 18.1, 17.7 Anal. Calcd. for C_6_H_11_NO_2_: C, 55.79; H, 8.57; N, 10.84. Found: C, 55.70; H, 8.49; N, 10.81.

*(4R, 5S)-(+)-4-methyl-5-phenyl-1,3-oxazolidin-2-one* (**2d**). The aminoalcohol **1d **(0.50 g, 3.30 mmol, 1.0 eq.), diethyl carbonate (0.58 g, 0.60 mL, 4.95 mmol, 1.5 eq.) and sodium methoxide (0.008 g, 0.16 mmol, 0.05 eq.) were placed in a 10 mL vessel in the microwave system and reacted under the BenchMate modality during 15 minutes at 135 °C and 145 W of power. When the reaction was completed, the resulting mixture was partitioned with a mixture of CH_2_Cl_2_ (20 mL) and H_2_O (20 mL). The organic layer was separated and the aqueous layer was extracted with CH_2_Cl_2_ (2 × 10 mL). The organic extracts were dried over Na_2_SO_4_ and the solvent evaporated to furnish a residue which was purified through a silica gel chromatographic column eluting with hexane-ethyl acetate (3:2) to give 0.50 g (87% yield) of **2d** as a white solid. The spectroscopic data were compared with those reported in the literature [9,10]: m. p. = 119–120 °C (Lit. = 121–123 °C), [α]_D_ = +162 c = 1.9 in CHCl_3_ ([α]_D(Lit.)_ = +168 c = 2 in CHCl_3_), e.e. = 96%. ^1^H-NMR (200 MHz, CDCl_3_): 7.40–7.26 (m, 5H), 6.25 (br, s, 1H), 5.71 (d, 1H, *J* = 8.0 Hz), 4.21 (m, 1H), 0.81 (d, 3H, *J *= 6.6 Hz). ^13^C-NMR (50 MHz, CDCl_3_): 159.6, 135.0, 128.6, 126.0, 81.2, 52.6, 17.8 Anal. Calcd. for C_10_H_11_NO_2_: C, 67.77; H, 6.20; N, 7.90. Found: C, 67.61; H, 6.18; N, 7.82.

### 3.3. Synthesis of Oxazolidine-2-thiones **3a-d**

In this case were used two different procedures. Compounds **3a** and **3b** were prepared using the same reagents and proportions reported by Wu. [16] Compounds **3c-d** were prepared according to the method described by LeCorre [17] with minor variations.

*(S)-4-Benzyl-1,3-oxazolidine-2-thione* (**3a**). In a 50 mL vessel provided with a condenser, the amino alcohol **1a **(1.00 g, 6.61 mmol, 1.0 eq.), K_2_CO_3_ (0.45 g, 3.30 mmol, 0.5 eq.), commercially available anhydrous ethanol (5 mL) and CS_2_ (1.00 g, 13.23 mmol, 0.8 mL, 2.0 eq.) were placed in the reactor under the Open Vessel modality with 50 °C and 50 W of power during 15 seconds. When the reaction system reached a 50 °C temperature, the microwave irradiation was stopped and a 35% H_2_O_2_ aq. solution (0.96 g, 9.92 mmol, 0.85 mL, 1.5 eq.) were added from the top of the condenser taking care the temperature of the reaction system did not overpassed from 50 °C. When the addition was completed, the insoluble K_2_SO_3_ were filtered off with suction. The filtrate was diluted with EtOAc (50 mL), washed with water (3 × 15 mL) and brine (3 × 15 mL). The organic layer was separated, dried over Na_2_SO_4_ and evaporated to yield a residue which was purified through a silica gel chromatographic column eluting with a hexane-ethyl acetate solvent system (6:4) to give 1.26 g of **3a** (99% yield) obtained as an oil. The spectroscopic data were compared with those reported in the literature [17]: [α]_D_ = −91.4 c = 1.87 in CHCl_3_ ([α]_D(Lit.)_ = −93.03 c= 1.88 in CHCl_3_), e.e. = 98%. ^1^H NMR (200 MHz, CDCl_3_): 7.98 (br, s, 1H), 7.39–7.15 (m, 5H), 4.68 (dd, 1H, *J* = 8.5, 8.5 Hz), 4.35 (m, 2H, *J* = 8.4 Hz), 2.92 (d, 2H, *J *= 6.6 Hz). ^13^C NMR (50 MHz, CDCl_3_): 189.5, 135.2, 129.2, 129.0, 127.6, 74.9, 58.0, 40.6 Anal. Calcd. for C_10_H_11_NOS: C, 62.19; H, 5.69; N, 7.25. Found: C, 61.92; H, 5.60; N, 7.23.

*(S)-4-Phenyl-1,3-oxazolidine-2-thione* (**3b**). In a 50 mL vessel provided with a condenser, the amino alcohol **1b** (1.0 g, 7.28 mmol, 1.0 eq.), K_2_CO_3_ (0.50 g, 3.64 mmol, 0.5 eq.), commercially available anhydrous ethanol (5 mL) and CS_2_ (1.11 g, 14.57 mmol, 0.9 mL, 2.0 eq.) were placed in the reactor under the Open Vessel modality with 50 °C and 50 W of power during 15 seconds. When the reaction system reached a 50 °C temperature, the microwave irradiation was stopped and a 35% H_2_O_2_ aq. solution (0.37 g, 10.92 mmol, 0.96 mL, 1.5 eq.) were added from the top of the condenser taking care the temperature of the reaction system did not overpassed from 50 °C. When the addition was completed, the insoluble K_2_SO_3_ were filtered off with suction. The filtrate was diluted with EtOAc (50 mL), washed with water (3 × 15 mL) and brine (3 × 15 mL). The organic layer was separated, dried over Na_2_SO_4_ and evaporated to yield a residue which was purified through a silica gel chromatographic column eluting with a hexane-ethyl acetate solvent system (6:4) to give 1.29 g of **3b** (99% yield) obtained as a slightly yellow solid. The spectroscopic data were compared with those reported in the literature [16,17]: m. p.= 118–120 °C, (Lit. = 121–122 °C), [α]_D_ = +76.02 c = 0.2 in CHCl_3_ ([α]_D(Lit.)_ = +77 c = 0.2 in CHCl_3_), o. p. = 97%. ^1^H NMR (200 MHz, CDCl_3_): 8.23 (br, s, 1H), 7.35–7.19 (m, 5H), 5.04 (dd, 1H, *J* = 6.6, 9.2 Hz), 4.92 (t, 1H, *J* = 9.2 Hz), 4.40 (dd, 1H, *J *= 6.6, 8.4 Hz). ^13^C NMR (50 MHz, CDCl_3_): 189.8, 138.0, 129.4, 129.2, 126.3, 77.8, 60.3 Anal. Calcd. for C_9_H_9_NOS: C, 60.30; H, 5.02; N, 7.81. Found: C, 60.27; H, 5.01; N, 7.69.

*(S)-4-Isopropyl-1,3-oxazolidine-2-thione* (**3c**). In a 25 mL vessel, the amino alcohol **1c **(0.50 g, 4.48 mmol, 1.0 eq.), K_2_CO_3_ (0.66 g, 4.48 mmol, 1.0 eq.) and CS_2_ (0.55 g, 7.27 mmol, 0.43 mL, 1.5 eq.) were placed in the reactor**. **The reaction mixture was stirred in the BenchMate modality with 50 °C and 50 W of power for 10 min. When the reaction was completed, the resulting mixture was partitioned with a mixture of CH_2_Cl_2_ (15 mL) and H_2_O (20 mL). The organic layer was separated and the aqueous layer was extracted with CH_2_Cl_2_ (2 × 15 mL). The organic extracts were dried over Na_2_SO_4_ and the solvent evaporated to furnish a residue which was purified through a silica gel chromatographic column eluting with a hexane-ethyl acetate solvent system (65:35) to give 0.57 g of **3c** (89% yield) obtained as a slightly yellow solid. The spectroscopic data were compared with those reported in the literature [17]: m. p. = 42–43 °C (Lit. = 45–46 °C), [α]_D_ = −20 c = 0.4 in CHCl_3_ ([α]_D(Lit.)_ = −22 c = 0.4 in CHCl_3_), e.e. = 90%. ^1^H NMR (200 MHz, CDCl_3_): 7.14 (br, s, 1H), 4.44 (t, 1H, *J* = 8.8 Hz), 4.09 (dd, 1H, *J* = 8.4, 5.9 Hz), 3.62 (dd, 1H, *J *= 15.0, 7.0 Hz), 1.71 (m, 1H), 0.96 (d, 3H, *J* = 6.6 Hz), 0.90 (d 3H, *J* = 6.6 Hz). ^13^C NMR (50 MHz, CDCl_3_): 160.7, 68.7, 58.5, 32.8, 18.1, 17.8 Anal. Calcd. for C_6_H_11_NOS: C, 49.67; H, 7.58; N, 9.65. Found: C, 49.50; H, 7.51; N, 9.53. 

*(4R, 5S)-(+)-4-methyl-5-phenyl-1,3-oxazolidine-2-thione *(***3d***). In a 25 mL vessel, the amino alcohol **1d **(0.5 g, 3.30 mmol, 1.0 eq.), K_2_CO_3_ (0.45 g, 3.30 mmol, 1.0 eq.) and CS_2_ (0.37g, 4.95 mmol, 0.3 mL, 1.5 eq.) were placed in the reactor**. **The reaction mixture was stirred in the BenchMate modality with 50 °C and 50 W of power for 10 min. When the reaction was over, the resulting mixture was partitioned with a mixture of CH_2_Cl_2_ (15 mL) and brine (20 mL). The organic layer was separated and the aqueous layer was extracted with CH_2_Cl_2_ (2 × 15 mL). The organic extracts were dried over Na_2_SO_4_ and the solvent evaporated. The product was purified by a silica gel chromatographic column eluting with a hexane-ethyl acetate solvent system (6:4) to give 0.52 g of **3d** (82% yield) obtained as a slightly yellow solid. The spectroscopic data were compared with those reported in the literature [17]. m. p. = 93–93 °C (Lit. = 95–97 °C), [α]_D_ = +215.01 c = 0.44 in CHCl_3_ ([α]_D(Lit.)_ = +219.2 c = 0.44 in CHCl_3_), e.e. = 98%. ^1^H NMR (200 MHz, CDCl_3_): 8.50 (br, s, 1H), 7.19–7.38 (m, 5H), 5.90 (d, 1H, *J* = 8.4 Hz), 4.44 (dq, 1H, *J* = 6.4, 8.4 Hz), 0.81 (d, 3H, *J *= 6.4 Hz). ^13^C NMR (50 MHz, CDCl_3_): 188.7, 133.4, 128.7, 128.3, 126.0, 87.9, 55.83, 15.9 Anal. Calcd. for C_10_H_11_NOS: C, 62.19; H, 5.69; N, 7.25. Found: C, 61.98; H, 5.52; N, 7.17.

### 3.4. Synthesis of Thiazolidine-2-thiones **4a-d**

These compounds were prepared according to the method described by LeCorre [17] without inorganic basic media.

*(S)-4-Benzyl-1,3-thiazolidine-2-thione *(**4a**). In a 10 mL reaction vessel were placed the amino alcohol **1a** (1.00 g, 6.61 mmol, 1.0 eq.), Et_3_N (1.67 g, 16.53 mmol, 2.3 mL, 2.5 eq.), CS_2_ (1.51 g, 19.84 mmol, 1.2 mL, 3.0 eq.) and DMSO (0.3 mL). Under the BenchMate modality **4a** was prepared in 60 minutes with 100 °C and 40 W of power. When the reaction was completed, 30 mL of water were added and the resulting mixture extracted with ethyl acetate. The organic layer was separated, dried over Na_2_SO_4_ and evaporated to yield a residue which was purified by a silica gel chromatographic column using a hexane-ethyl acetate (7:3) solvent system to give 1.27 g of **4a** (92% yield) obtained as a slightly yellow solid. The spectroscopic data were compared with those reported in the literature [17]: m. p. = 82–83 °C (Lit. = 84–85 °C), [α]_D_ = −120.01 c = 0.96 in CHCl_3_ ([α]_D(Lit.)_ = −122 c = 1 in CHCl_3_), e.e. = 98%. ^1^H NMR (200 MHz, CDCl_3_): 8.01 (br, s, 1H), 7.41–7.17 (m, 5H), 4.45 (q, 1H, *J* = 7.2 Hz), 3.61 (dd, 1H, *J* = 11.2, 7.7 Hz), 3.33 (dd, 1H, *J* = 11.0, 7.0 Hz), 3.0 (d, 2H, *J* = 7.4 Hz). ^13^C NMR (50 MHz, CDCl_3_): 201.0, 135.9, 129.3, 129.1, 127.6, 65.2, 40.4, 38.4 Anal. Calcd. for C_10_H_11_NS_2_: C, 57.37; H, 5.25; N, 6.68. Found: C, 57.25; H, 5.20; N, 6.54.

*(S)-4-Phenyl-1,3-thiazolidine-2-thione *(**4b**). In a 10 mL reaction vessel were placed the amino alcohol **1b** (1.00 g, 7.29 mmol, 1.0 eq.), Et_3_N (1.84 g, 18.22 mmol, 2.5 mL, 2.5 eq.), CS_2_ (1.66 g, 21.87 mmol, 1.3 mL, 3.0 eq.) and DMSO (0.3 mL). Under the BenchMate modality **4b** was prepared in 90 minutes with 100 °C and 40 W of power. When the reaction was completed, 50 mL of water were added and the resulting mixture extracted with ethyl acetate. The organic layer was separated, dried over Na_2_SO_4_ and evaporated to yield a residue which was purified by a silica gel chromatographic column using a hexane-ethyl acetate (6:4) solvent system to give 1.26 g of **4b** (89% yield) obtained as a yellow solid. The spectroscopic data were compared with those reported in the literature [17]: m. p. = 122–123 °C (Lit. = 125–126 °C), [α]_D_ = +205.03 c = 0.9 in CHCl_3_ ([α]_D(Lit.)_ = +207 c = 1 in CHCl_3_), e.e. = 99%. ^1^H NMR (200 MHz, CDCl_3_): 8.07 (br, s, 1H), 7.41–7.35 (m, 5H), 5.32 (t, 1H, *J* = 8.1 Hz), 3.84 (dd, 1H, *J* = 11.0, 8.0 Hz), 3.48 (dd, 1H, *J *= 11.0, 8.0 Hz). ^13^C NMR (50 MHz, CDCl_3_): 201.0, 137.4, 128.8, 128.7, 125.7, 66.9, 44.0 Anal. Calcd. for C_9_H_9_NS_2_: C, 55.34; H, 4.60; N, 7.16. Found: C, 55.20; H, 4.51; N, 7.08.

*(S)-4-Isopropyl-1,3-thiazolidine-2-thione* (**4c**). In a 10 mL reaction vessel were placed the amino alcohol **1c** (0.50 g, 4.84 mmol, 1.0 eq.), Et_3_N (1.22 g, 12.11 mmol, 1.7 mL, 2.5 eq.), CS_2_ (1.10 g, 14.54 mmol, 0.9 mL, 3.0 eq.) and DMSO (0.3 mL). Under the BenchMate modality **4c** was prepared in 110 minutes with 110 °C and 100 W of power. When the reaction was completed, 40 mL of water were added and the resulting mixture extracted with ethyl acetate. The organic layer was separated, dried over Na_2_SO_4_ and evaporated to yield a residue which was purified by a silica gel chromatographic column using a hexane-ethyl acetate (6:4) solvent system to give 0.70 g of **4c** (90% yield) obtained as a yellow solid. The spectroscopic data were compared with those reported in the literature [17]: m. p. = 67–68 °C (Lit. = 69–71 °C), [α]_D_ = −35.1 c = 1 in CHCl_3_ ([α]_D(Lit.)_ = −37 c = 1 in CHCl_3_), e.e. = 94%. ^1^H NMR (200 MHz, CDCl_3_): 8.54 (br, s, 1H), 4.09 (m, 1H), 3.50 (dd, 1H, *J* = 7.9, 10.8 Hz), 3.24 (dd, 1H, *J* = 7.9, 10.8 Hz), 1.97 (m, 1H), 0.99 (d, 3H, *J* = 7.0 Hz), 0.97 (d, 3H, *J* = 7.0 Hz). ^13^C NMR (50 MHz, CDCl_3_): 200.5, 70.1, 35.5, 31.7, 18.5, 18.2 Anal. Calcd. for C_6_H_11_NS_2_: C, 44.67; H, 6.82; N, 8.68. Found: C, 44.60; H, 6.87; N, 8.58.

*(4R, 5R)-(+)-4-methyl-5-phenyl-1,3-thiazolidine-2-thione *(**4d**). In a 10 mL reaction vessel were placed the amino alcohol **1d** (0.50 g, 3.30 mmol, 1.0 eq.), Et_3_N (0.83 g, 8.26 mmol, 1.15 mL, 2.5 eq.), CS_2_ (0.75 g, 9.91 mmol, 0.6 mL, 3.0 eq.) and DMSO (0.3 mL). Under the BenchMate modality **4d** was prepared in 120 minutes with 110 °C and 100 W of power. When the reaction was completed, 40 mL of water were added and the resulting mixture extracted with ethyl acetate. The organic layer was separated, dried over Na_2_SO_4_ and evaporated to yield a residue which was purified by a silica gel chromatographic column using a hexane-ethyl acetate (6:4) solvent system to give 0.45 g of **4d** (65% yield) obtained as a yellow solid. The spectroscopic data were compared with those reported in the literature [17,19,20,21,22,23,24]: m. p. = 72–73 °C (Lit. = 75–77 °C), [α]_D_ = +30.7 c = 1 in CHCl_3_ ([α]_D(Lit.)_ = +33 c = 1 in CHCl_3_), e.e. = 93%. ^1^H NMR (200 MHz, CDCl_3_): 7.89 (br, s, 1H), 7.45–7.22 (m, 5H), 4.68 (d, 1H, *J* = 8.8 Hz), 4.01 (m, 1H), 1.43 (d, 3H, *J* = 6.6 Hz). ^13^C NMR (50 MHz, CDCl_3_): 199.1, 136.9, 129.3, 129.1, 128.1, 74.8, 53.2, 18.5 Anal. Calcd. for C_10_H_11_NS_2_: C, 57.32; H, 4.78; N, 6.69. Found: C, 57.32; H, 4.74; N, 6.50.

## 4. Conclusions

In summary, the present work reports a practical synthesis of oxazolidin-2-ones, oxazolidine-2-thiones and thiazolidine-2-thiones, chiral auxiliaries widely used in asymmetric synthesis, under microwave conditions, with a remarkable reduction in the reactions times required to achieve their synthesis and with improved yields, particularly in the case of the thiazolidine-2-thiones.
